# Development of Extracellular Matrix-Retaining Mesenchymal Stromal Cell Fibers for Novel Endovascular Regenerative Therapy for Aortic Disease

**DOI:** 10.3390/jfb17040165

**Published:** 2026-04-01

**Authors:** Soichiro Fukushima, Lupeng Teng, Makoto Koizumi, Minami Hasegawa-Ogawa, Hiroki Ohta, Ryosuke Iwai, Hirotaka James Okano, Takao Ohki

**Affiliations:** 1 Division of Vascular Surgery, Department of Surgery, The Jikei University School of Medicine, 3-25-8 Nishi-Shinbashi, Minato-ku, Tokyo 105-8461, Japan; sfuku@jikei.ac.jp; 2 Division of Regenerative Medicine, Research Center for Medical Sciences, The Jikei University School of Medicine, 3-25-8 Nishi-Shinbashi, Minato-ku, Tokyo 105-8461, Japan; koizumim@jikei.ac.jp (M.K.); m.hasegawa@jikei.ac.jp (M.H.-O.); hiro-o@jikei.ac.jp (H.O.); hjokano@jikei.ac.jp (H.J.O.); 3 Division of Natural Science, Graduate School of Science and Engineering, Okayama University of Science, 1-1 Ridaicho, Kita-ku, Okayama 700-0005, Japan; r23ndv8iu@ous.jp; 4 Institute of Frontier Science and Technology, Okayama University of Science, 1-1 Ridaicho, Kita-ku, Okayama 700-0005, Japan

**Keywords:** aortic aneurysm, endovascular treatment, tissue engineering, cell self-aggregation technique, mesenchymal stromal cell, regenerative therapy

## Abstract

Postoperative aneurysm sac enlargement is a significant clinical issue in endovascular aortic aneurysm repair that is potentially associated with impaired microcirculation in the aneurysmal wall. We developed centimeter-long, fiber-shaped aggregates of human bone-marrow-derived mesenchymal stromal cells (HMSC fiber) to function as a scaffold-free cellular construct applicable to endovascular treatment. HMSC fibers were prepared using a cell self-aggregation technique and optimized by controlling the cell number per unit length to preserve cellular viability and mechanical stability. The resulting fibers retained mesenchymal stromal cell characteristics and endogenous extracellular matrix, facilitating smooth handling and intraluminal delivery without structural collapse. After transcatheter administration into a swine aortic aneurysm model, HMSC fiber-induced fibroconnective tissue formation occurred with capillary-like structures within the aneurysm sac. These findings demonstrate the feasibility of HMSC fiber as a controllable and stable platform for localized endovascular cell delivery. Furthermore, this study established their potential utility as a regenerative adjunct to current endovascular treatment for aortic disease.

## 1. Introduction

Postoperative aneurysm sac poses a significant concern following endovascular aortic aneurysm repair (EVAR). The EVAR trial-1, which prospectively followed patients who underwent abdominal aortic aneurysm for 15 years postoperatively, found that secondary aneurysm rupture due to aneurysm enlargement occurred in the EVAR group [1]. Microcirculatory disorder within the aneurysm wall, specifically, impaired vasa vasorum perfusion, may cause aneurysm wall enlargement [2]. Within the aortic wall, the inner third is perfused by lumen-side blood flow, whereas the outer two-thirds rely on the vasa vasorum for blood supply [2]. Endovascular treatment with a stent graft (SG) disrupts lumen-side perfusion. Consequently, the aortic wall inevitably becomes solely dependent on perfusion from the adventitial vasa vasorum. Thus, we propose that mitigating the microcirculatory impairment of the vasa vasorum is essential for preventing aneurysm enlargement after SG repair.

**Figure 1:**
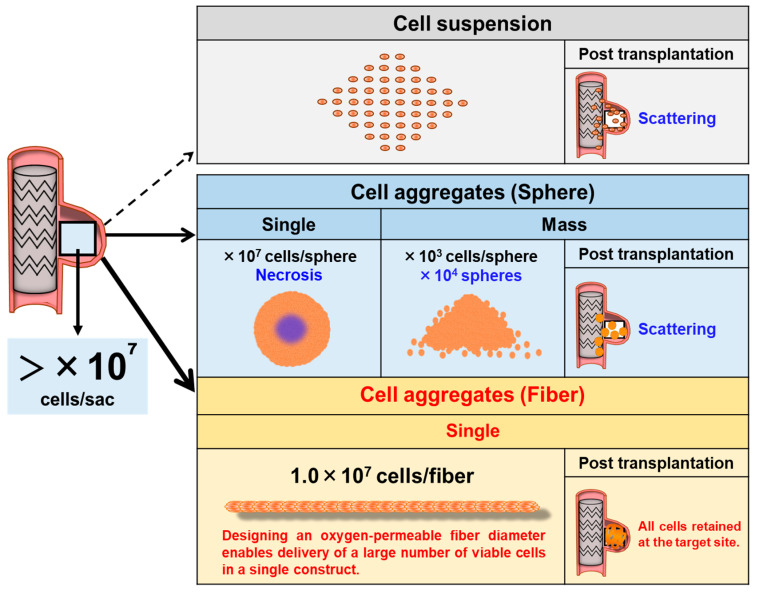
Modes of cell transplantation into the aneurysm sac following endovascular stent graft repair. This schematic compares single-cell suspension and spheroid delivery with the transplantation of fiber-shaped cell aggregates designed to improve localized intraluminal cell retention.

A regenerative approach involving the direct administration of human mesenchymal stromal cells (HMSCs) to the aneurysm sac has emerged as a promising option. HMSCs may promote aneurysm wall healing through several mechanisms: (1) suppression of inflammation via anti-inflammatory cytokines; (2) promotion of angiogenesis and extracellular matrix (ECM) organization through paracrine signaling; and (3) reconstruction of medial layers via differentiation into vascular smooth muscle cell-like phenotypes [3,4,5,6]. From a quantitative perspective, regeneration of aneurysmal tissue volume in the order of several cubic centimeters would require approximately 10^7^–10^8^ HMSCs, based on estimations of cellular volume and packing density. However, HMSC suspensions administered by direct injection are poorly retained and rapidly washed out under arterial blood flow (as observed in our preliminary investigations), leading to insufficient engraftment for therapeutic use (Figure 1).

Although the delivery of spheroid-based HMSCs can improve cell survival and paracrine activity [7], MSC spheroids are prone to developing central hypoxia and necrosis in a size-dependent manner, which may compromise cell viability and therapeutic function [8,9]. Furthermore, when numerous spheroids are administered, they tend to disperse within the aneurysm sac rather than remain concentrated in the target region, indicating insufficient localized cell retention (Figure 1). This limitation mirrors that of single-cell suspension delivery, in which cells are similarly scattered and rapidly washed out under blood flow. Consequently, a catheter-deliverable cellular construct with structural stability and in situ retention capability must be developed to achieve endovascular regenerative therapy. To address this challenge, we employed the cell self-aggregation technique (CAT), a proprietary scaffold-free tissue fabrication method developed by our group [10]. CAT rapidly forms three-dimensional cellular constructs through patterned self-aggregation, which includes spheroids [11,12], rings [13,14], fibers [15,16], and band-shaped tissues [17] to develop multiple scaffold-free formats for regenerative applications. Especially designed for endovascular use, CAT enables the generation of contrast-agent-loaded HMSC aggregates suitable for radiographic visualization and catheter delivery [16], making this platform feasible for regenerative catheter-based applications. Building on this platform, we developed fiber-shaped multicellular aggregate composed of HMSC (HMSC fiber) as a catheter-deliverable, structurally stable alternative to spheroids. HMSC fibers retain strong cell–cell interactions while allowing oxygen and nutrient diffusion along the fiber axis, thereby reducing central necrosis (Figure 1). Moreover, they can be aspirated, delivered, and positioned through catheters as a single continuous unit, minimizing uncontrolled dispersion and facilitating stable placement. Thus, HMSC fibers present a promising regenerative modality for definitive aneurysm wall reconstruction following endovascular treatment.

In this study, we investigated the preparation and biological characteristics of CAT-derived HMSC fibers and evaluated their feasibility for catheter-mediated intraluminal transplantation into aortic aneurysm models following endovascular treatment using SGs. We sought to establish foundational evidence supporting HMSC fiber as a novel regenerative platform for aneurysm wall healing following endovascular repair.

## 2. Materials and Methods

### 2.1. Establishment of the Swine Thoracic Aortic Aneurysm Model

**Figure 2:**
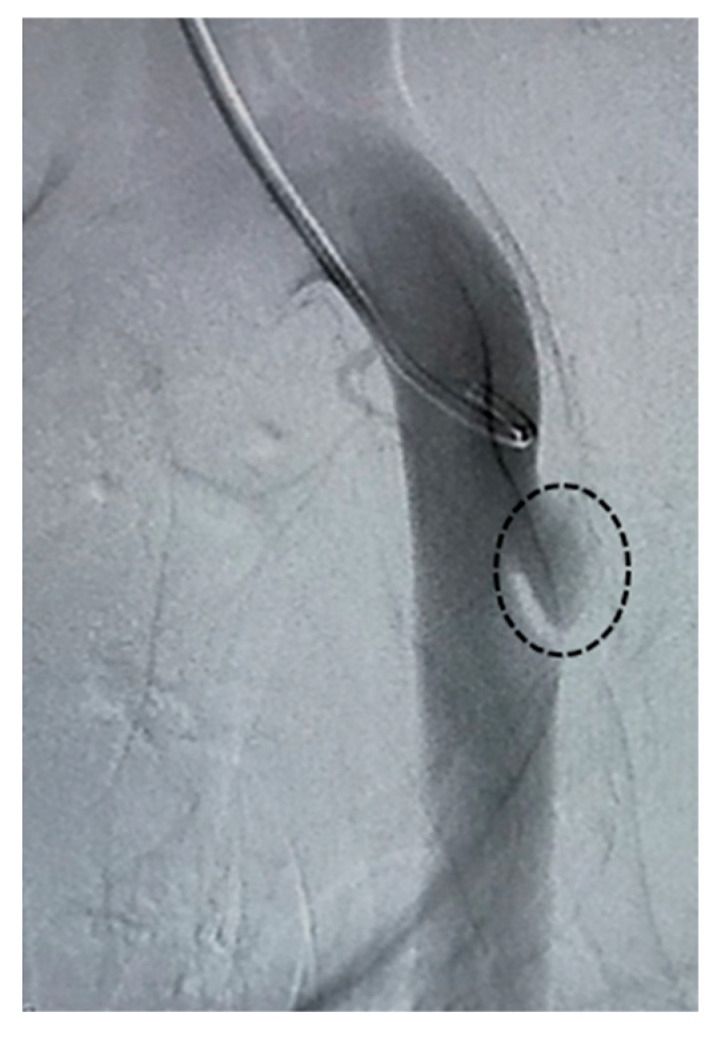
Angiographic image of the swine thoracic aortic aneurysm (TAA) model. Dashed circles delineate the TAA.

This animal experimental study was approved by the Institutional Animal Care and Use Committee of The Jikei University School of Medicine (IRB number: 2019-020). Previously, we developed a method of establishing a swine thoracic aortic aneurysm (TAA) model [18]. This study also employed the same technique in swine weighing 35–40 kg. All animals underwent the same model induction procedures under sedation with subcutaneous medetomidine injection and general anesthesia with inhaled isoflurane. The animals were placed in the right lateral position, and left thoracotomy was performed to surgically expose the thoracic aorta. Heparin 4000 U was administered, followed by aortic clamping. A surgical incision was made around the anterior wall of the exposed aorta. A subintimal space was bluntly dissected at the incision site to create an aneurysm space. After suture closure for the adventitia, aortic clamping was released. The incision site was irrigated, hemostasis was confirmed, and the wound was closed. Fluoroquinolone was administered for wound prophylaxis. Levofloxacin 75 mg/day was administered orally for wound infection prophylaxis for 1 week postoperatively. Two weeks after model creation, angiography was performed under general anesthesia. After confirming the establishment of the TAA (Figure 2), the model animals underwent endovascular repair. HMSC fiber therapy or saline was administered selectively into the target aneurysm sac through a catheter.

### 2.2. Cell Culture and HMSC Fiber Preparation

**Figure 3:**
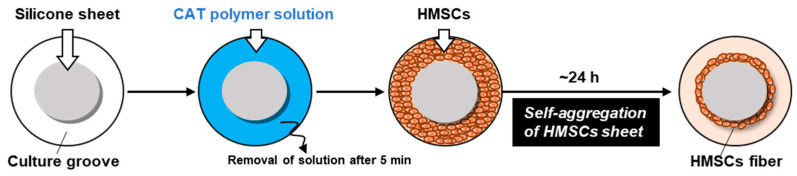
Schematic illustration of the preparation of human mesenchymal stromal cell (HMSC) fiber using the cell self-aggregation technique (CAT)-based self-aggregation method.

Bone-marrow-derived HMSCs were purchased from Promocell (Heidelberg, Germany) and cultured in mesenchymal stem cell growth medium 2 with 10% supplement (Promocell) and 1% penicillin–streptomycin at 37 °C and 5% CO_2_. Third- or fourth-passage HMSCs were used for HMSC fiber preparation. HMSC fibers were prepared using a ring-shaped culture groove using the CAT (Figure 3), as described previously [16]. In detail, a 2 mm thick silicone sheet (Togawa Rubber Co., Ltd., Osaka, Japan) was cut into a 60 mm diameter disk and fixed to the surface of a 100 mm diameter culture dish (AGC Techno Glass Co., Ltd., Shizuoka, Japan), thereby creating an annular culture groove in the peripheral region of the dish. The CAT polymer, poly(2-(dimethylamino) ethyl methacrylate)-*co*-poly(methacrylic acid) (supplied by Nissan Chemical Corporation, Tokyo, Japan), was dissolved in UltraPure Water (Thermo Fisher Scientific Inc., Waltham, MA, USA) at a concentration of 100 μg/mL, as described previously [10]. A 5 mL portion of the polymer solution was poured into the culture groove and left to stand at room temperature for 5 min. Subsequently, the excess solution was removed by aspiration to produce a self-aggregation-inducing surface. HMSCs were seeded into the polymer-coated groove at a cell number of 4 × 10^6^ to 12 × 10^6^ cells/cm^2^ and cultured overnight. During culture, the cells first formed a confluent monolayer sheet and subsequently underwent spontaneous self-aggregation, forming a ring-shaped cell aggregate along the groove (Figure 3). Following culture completion, the silicone disk was removed using a forceps, and the ring-shaped aggregate was cut at a single point with scissors to produce one piece of HMSC fiber.

### 2.3. Endovascular HMSC Fiber Treatment

The pre-established swine TAA model animals (total *n* = 5) were assigned to two intervention groups without formal randomization: a control group (*n* = 2) undergoing SG placement followed by saline infusion, and an HMSC fiber group (*n* = 3) undergoing SG placement followed by intraluminal transplantation of HMSC fibers. No animals were excluded after the procedure. Under isoflurane inhalation anesthesia, a 5-Fr sheath (Boston Scientific, Marlborough, MA, USA) was inserted retrogradely through the right common carotid artery (CCA). Next, a pigtail catheter (TERUMO Medical Corporation, Somerset, NJ, USA) was positioned in the ascending aorta for angiography, performed to confirm the presence of the TAA. Thereafter, to facilitate SG delivery and placement, the abdominal aorta was surgically exposed through a midline abdominal incision.

First, a 5-Fr sheath was inserted into the abdominal aorta, after which an Amplatz super stiff guidewire (Boston Scientific Japan, Tokyo, Japan) was advanced into the ascending aorta. The 5-Fr sheath was then replaced with a 12-Fr DrySeal sheath (W. L. Gore & Associates, Inc., Flagstaff, AZ, USA) for SG delivery. We used the Excluder iliac extender (W. L. Gore & Associates, Inc.) as a treatment device, which has a zero-porosity structure and is clinically used in the abdominal–iliac artery region; its diameter is compatible with the normal swine thoracic aorta (approximately 13–14 mm). In accordance with clinical practice, a sealing length of 2 cm was secured on either the proximal or distal side of the aorta during SG placement. Prior to SG deployment, a 4-Fr angiographic catheter (Tempo^®^, Miami Lakes, FL, USA) was preplaced into the aneurysm sac via a 5-Fr sheath inserted through the right CCA. The HMSC fibers used for transplantation were prepared approximately 1 day prior to the procedure and maintained in culture medium until use. After deploying the SG, either HMSC fiber or an equal volume (0.7 mL) of saline was administered into the aneurysm sac through the catheter. After administration, the infusion catheter was removed promptly from the aneurysm space. After confirmation of accurate SG deployment by angiography, both sheaths inserted into the right CCA and abdominal aorta were removed, and the access site was closed using 6-0 Prolene sutures. Following wound closure, fluoroquinolone topical medication was applied to the neck and abdominal incision sites, which were then covered with gauze dressings to prevent contamination. Postoperatively, oral levofloxacin (75 mg/day) was administered for 7 days for infection control.

### 2.4. Follow-Up Angiography

Two weeks after the treatment, follow-up angiography was performed under general anesthesia with isoflurane inhalation. First, a 5-Fr sheath was inserted through the femoral artery. After confirming the absence of an endoleak for the aneurysm and the absence of complications, such as thrombosis or embolism in any organs, the animal was euthanized via intravenous administration of 20 mg potassium chloride according to the animal experiment protocol. The thoracic aorta was harvested, preserved in 4% paraformaldehyde, and evaluated by histological analysis.

### 2.5. Histological Analysis

HMSC fibers prepared at defined cell numbers per unit length were fixed in 4% paraformaldehyde, embedded in paraffin, and sectioned at 4 to 5 μm thickness. Consecutive sections were stained routinely with hematoxylin–eosin (HE) and Masson’s trichrome stain. For immunofluorescence analysis, sections were deparaffinized and rehydrated following standard procedures. Antigen retrieval was performed by immersing the sections in an antigen activator immunosaver (Nisshin-EM, Tokyo, Japan) heated to 90 °C for 40 min. Blocking was performed using 1% bovine serum albumin in phosphate-buffered saline (PBS) (−) at room temperature for 1 h. The sections were incubated with primary antibodies of anti-fibronectin mouse monoclonal (1:100; ab6328; Abcam, Cambridge, UK), anti-CD44 mouse monoclonal (1:100; ab6124; Abcam), anti-CD105 rabbit polyclonal (1:100; ab107595; Abcam), and anti-vascular endothelial growth factor (VEGF) rabbit polyclonal (1:50; 19003-1-AP; Proteintech, IL, USA) at 4 °C for 15 h, followed by secondary antibodies of rabbit anti-mouse IgG H&L (Alexa Fluor^®^ 594; 1:1000; ab150128; Abcam) for fibronectin and CD44 and goat anti-rabbit IgG H&L (Alexa Fluor^®^ 594; 1:1000; ab150080; Abcam) for CD105, and goat anti-rabbit IgG H&L (Alexa Fluor^®^ 488; 1:1000; ab150077; Abcam) for VEGF at room temperature for 2 h. The sections were mounted on the ProLong Diamond Antifade Mountant with 4′,6-diamidino-2-phenylindole (P36971; Thermo Fisher Scientific) for nuclear staining. The sections were observed under an inverted fluorescence microscope (Eclipse Ti2; Nikon Corporation, Tokyo, Japan) equipped with a camera (DS-Fi3; Nikon corporation) and proprietary imaging software (NIS-Elements VERSION 5.20.00; Nikon corporation, Tokyo, Japan). The fluorescence colors shown in the images were assigned as pseudocolors using imaging software. Aneurysm specimens harvested 2 weeks after HMSC fiber transplantation were fixed in 4% paraformaldehyde, embedded in paraffin, and sectioned at 4 to 5 μm thickness. Serial sections were stained with Masson’s trichrome stain. Immunofluorescence staining was performed by incubating the sections with antibodies of anti-α-smooth muscle actin (α-SMA) mouse monoclonal (1:100; 904601; BioLegend, San Diego, CA, USA), anti-CD31 rabbit polyclonal (1:50; ab28364; Abcam), and anti-vimentin mouse monoclonal (1:200; ab8069; Abcam). Subsequently, the sections were incubated with secondary antibodies of rabbit anti-mouse IgG H&L (Alexa Fluor^®^ 594; 1:1000; ab150128; Abcam) for α-SMA and goat anti-rabbit IgG H&L (Alexa Fluor^®^ 488; 1:1000; ab150077; Abcam) for CD31, and donkey anti-mouse IgG H&L (Alexa Fluor^®^ 594; 1:1000; ab150108; Abcam) for vimentin at room temperature for 2 h. Fluorescence images were obtained using the same aforementioned microscope system.

### 2.6. Cell Viability Assay

Cell viability of HMSC fibers prepared at cell numbers per unit length of 50 × 10^4^ to 200 × 10^4^ cells/cm was evaluated using a Cell Proliferation Kit I (MTT) (Roche, Basel, Switzerland) according to the manufacturer’s protocol with minor modifications. Briefly, HMSC fibers formed approximately 24 h after cell seeding were collected and washed with phosphate-buffered saline (PBS) (−). The fibers were then cut into fragments of adjusted length so that each fragment contained approximately 3.1 × 10^5^ cells, and transferred into 48-well plates (AGC Techno Glass Co., Ltd.) containing 200 μL of culture medium per well. Subsequently, 20 μL of MTT labeling reagent was added to each well and incubated for 4 h at 37 °C in a humidified atmosphere with 5% CO_2_. After incubation, 200 μL of solubilization buffer was added to each well and incubated overnight under the same conditions to dissolve the fiber fragments. The supernatant was then transferred to 96-well plates (AGC Techno Glass Co., Ltd.), and the absorbance at 550 nm was measured using a microplate reader. Cell viability was calculated as a relative value, with the mean absorbance of fibers prepared at 50 × 10^4^ cells/cm defined as 100%. Five independent samples were analyzed for each group (*n* = 5).

### 2.7. Statistical Analysis

Quantitative data are presented as mean ± standard deviation. Statistical comparisons between groups were performed using Student’s *t*-test. Differences were considered statistically significant at *p* < 0.05.

## 3. Results

### 3.1. Preparation and Characteristics of the HMSC Fibers

**Figure 4:**
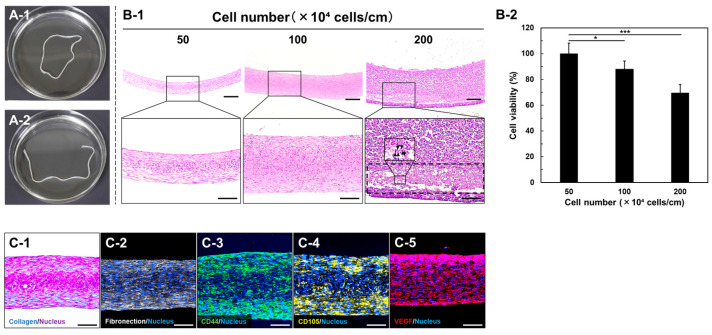
Formation and characterization of human mesenchymal stromal cell (HMSC) fibers. Gross images of a ring-shaped MSC aggregate formed at a cell density of 100 × 10^4^ cells/cm around a silicone disk (**A-1**) and the resulting HMSC fiber obtained by cutting the aggregate at a single point (**A-2**). Hematoxylin–eosin-stained longitudinal sections of HMSC fibers prepared at different cell numbers per unit length (**B-1**). Dashed boxes indicate regions of cellular degradation; arrows indicate nuclear debris. Scale bars: upper images, 200 μm; lower images, 100 μm. Quantitative analysis of cell viability in HMSC fibers prepared at different cell numbers per unit length (**B-2**). Data are presented as mean ± standard deviation (*n* = 5). * *p* < 0.05; *** *p* < 0.001. Masson’s trichrome-stained longitudinal sections of HMSC fibers prepared at a cell density of 100 × 10^4^ cells/cm (**C-1**). Immunofluorescence images for fibronectin (**C-2**), CD44 (**C-3**), CD105 (**C-4**), and VEGF (**C-5**). Scale bar: 100 μm.

**Figure 5:**
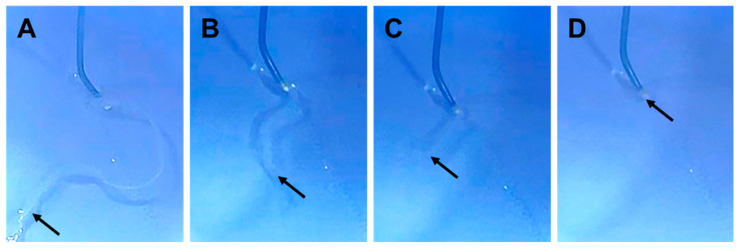
Catheter-based handling of human mesenchymal stromal cell (HMSC) fiber. Sequential images showing the process of loading an HMSC fiber into a catheter lumen. The HMSC fiber was aligned at the catheter tip (**A**), subsequently accommodated within the catheter lumen (**B**,**C**), and fully contained inside the catheter prior to delivery (**D**). Arrows indicate the tip region of the HMSC fiber.

Within approximately 1 h of culture, the HMSCs seeded into the ring-shaped culture groove for HMSC fiber preparation formed a confluent cell monolayer sheet without intercellular gaps, a result consistent with our previous report [16]. Subsequently, the cell sheet detached from the culture surface from the outer edge toward the inner region and underwent spontaneous aggregation, forming a ring-shaped HMSC aggregate around the silicone disk at 1 day after seeding. When the silicone disk was detached from the culture surface, the ring-shaped HMSC aggregate floated freely in the culture medium (Figure 4A-1). By cutting the aggregate at a single point, a fiber-shaped HMSC aggregate with a length of approximately 18 cm, demarcated by the circumference of the silicone disk, was obtained as the HMSC fibers (Figure 4A-2). The resulting HMSC fibers could be lifted and handled with forceps without rupture (Supplementary Video S1). When the number of seeded cells was adjusted to achieve cell numbers per unit length ranging from 50 × 10^4^ to 200 × 10^4^ cells/cm, HMSC fibers were successfully prepared irrespective of the cell number per unit length (Figure 4B). Histological analysis of longitudinal sections revealed that the thickness of HMSC fibers increased with cell number per unit length, reaching a length of approximately 100–500 μm across this range (Figure 4B-1). Notably, HMSC fibers prepared at 200 × 10^4^ cells/cm contained internal regions with indistinct and fragmented nuclei (dashed boxes and arrows), indicating the occurrence of cell necrosis likely caused by insufficient oxygen and nutrient supply. Consistent with these histological observations, quantitative analysis of cell viability demonstrated a progressive decrease in viable cells as the cell number per unit length increased. Compared with fibers prepared at 50 × 10^4^ cells/cm, cell viability decreased by approximately 10–15% at 100 × 10^4^ cells/cm and by about 30% at 200 × 10^4^ cells/cm (Figure 4B-2). For biological characterization, HMSC fibers prepared at a cell number per unit length of 100 × 10^4^ cells/cm were subjected to histological and immunohistochemical analyses. Masson’s trichrome and immunofluorescence staining for ECM components (collagen and fibronectin; Figure 4C-1,C-2) and mesenchymal cell markers (CD44 and CD105; Figure 4C-3,C-4) and the angiogenic growth factor VEGF (Figure 4C-5) revealed the widespread distribution of these markers throughout the HMSC fibers. These results indicate that most HMSCs constituting the fiber structure retained MSC characteristics while being surrounded by ECM.

### 3.2. HMSC Fiber Manipulation

When negative pressure was applied to the catheter, the HMSC fiber was smoothly aspirated from the catheter tip without structural collapse and was completely accommodated within the catheter lumen (Figure 5A and Supplementary Video S2). The HMSC fiber remained intact during aspiration and was stored completely inside the catheter along its entire length (Figure 5B,D).

### 3.3. Transplantation

**Figure 6:**
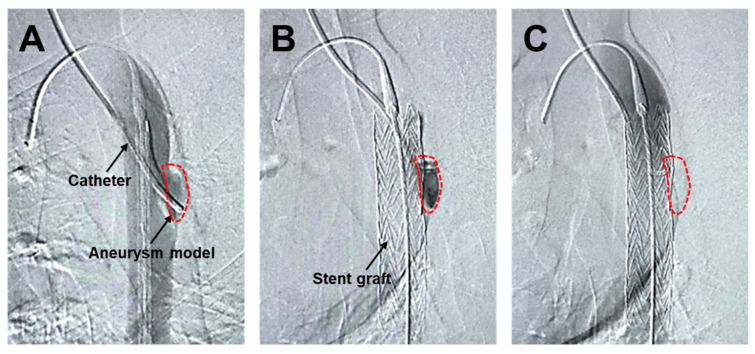
Angiographic assessment of human mesenchymal stromal cell (HMSC) fiber delivery using a stent graft. Angiographic imaging of the aneurysm model with a catheter prepositioned within the aneurysm sac (demarcated by the dashed line) (**A**). SG deployment covering the aneurysm while maintaining the catheter position within the aneurysm sac, enabling the administration of the HMSC fibers under reduced blood washout conditions (**B**). Follow-up angiography using a pigtail catheter after the procedure, confirming sustained aneurysm sealing (**C**).

The angiographic catheter prepositioned within the aneurysm model (Figure 6A) was maintained in situ during and after SG deployment (Figure 6B), creating a condition that minimized intraluminal blood washout within the aneurysm sac. Under these conditions, the HMSC fiber was transplanted through the catheter and into the aneurysm sac. Although the HMSC fiber itself was not directly visualized by angiography, no residual fibers were observed within the catheter lumen, and the catheter was removed smoothly without resistance. Furthermore, follow-up angiography performed using a pigtail catheter demonstrated sustained aneurysm sealing after the procedure (Figure 6C). Collectively, these observations are consistent with the successful intraluminal delivery of the HMSC fiber into the aneurysm sac.

**Figure 7:**
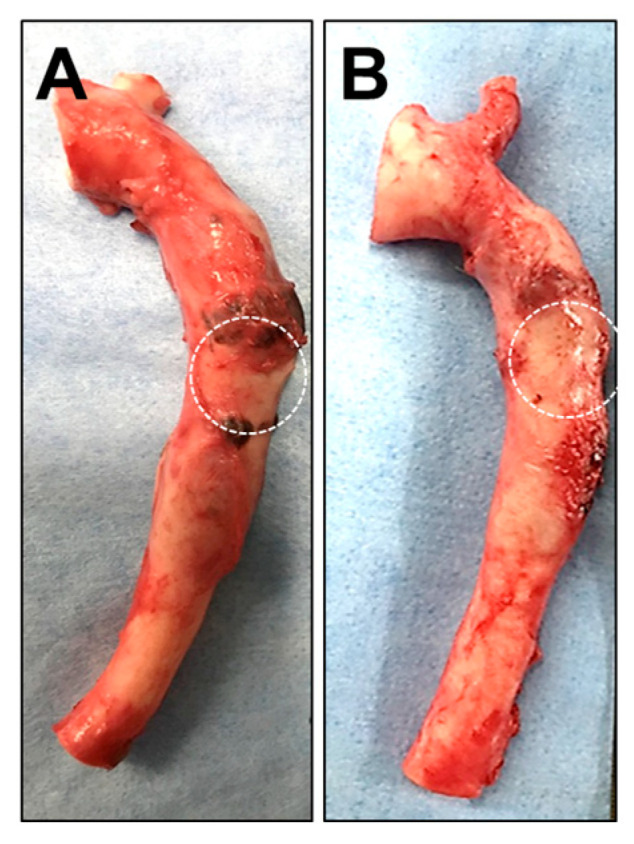
Macroscopic appearance of thoracic aorta segments with and without human mesenchymal stromal cell (HMSC) fiber administration. Representative gross images of thoracic aorta specimens transplanted into the aneurysm model without (**A**) or with (**B**) HMSC fiber administration. The dashed circles indicate the aneurysm region.

Two weeks after the administration of either saline or the HMSC fiber, the thoracic aorta specimens were harvested and grossly examined. The images shown represent representative findings observed among the animals in each group. No significant macroscopic differences were observed in the aneurysm region between the two intervention groups (Figure 7A,B), indicating that any potential effects of the interventions were not readily detectable by gross inspection at this time point.

**Figure 8:**
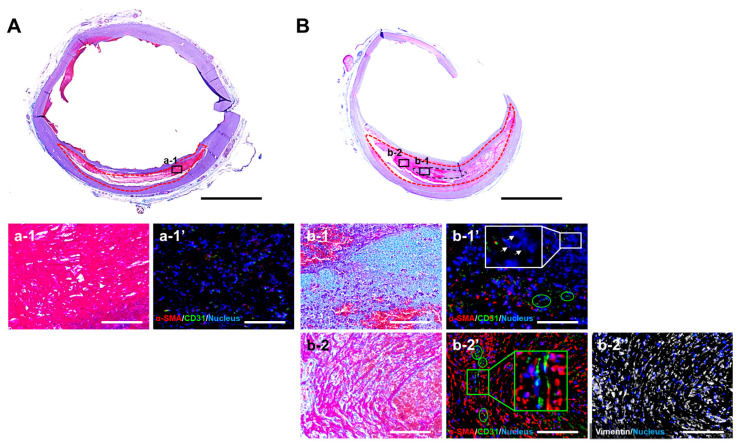
Masson’s trichrome-stained cross-sectional images of the swine aortic aneurysm model 2 weeks after without (**A**) or with human mesenchymal stromal cell (HMSC) fiber transplantation (**B**). Higher magnification images of thrombus-accumulated regions in aneurysms without (**a-1**) or with HMSC fiber transplantation (**b-1**, **b-2**). Enlarged views of Masson’s trichrome-stained specimens (**a-1**, **b-1**, and **b-2**); immunofluorescence staining for alfa-smooth muscle actin (α-SMA), a marker of myofibroblast-like cells, and CD31, an endothelial cell marker (**a-1′**, **b-1′**, and **b-2′**), and vimentin, a mesenchymal cell marker (**b-2″**). The red dashed outline indicates the aneurysmal segment, while the black dashed outline marks the transplanted HMSC fiber. Green circles and box indicate capillary-like structures; white arrows indicate fragmented nuclei. Black and white scale bars indicate 1 mm and 100 μm, respectively.

Masson’s trichrome staining of cross-sectional slices of the aneurysm model revealed the formation of a reddish-purple thrombus within the aneurysmal lumen (red dashed outline), regardless of whether HMSC fibers were transplanted (Figure 8A,B). At higher magnification, aneurysms without HMSC fiber transplantation contained only a thrombus and exhibited minimal additional tissue formation (Figure 8a-1). In contrast, in aneurysms transplanted with HMSC fibers, a thin, light-blue region with high cellular density was observed within the thrombus (Figure 8b-1), indicating tissue derived from the transplanted HMSC fiber (black dashed outline). Furthermore, a region located several millimeters away from the HMSC fiber contained areas within the thrombus showing very faint, minimal collagen deposition (Figure 8b-2). Immunofluorescence analysis revealed abundant α-SMA-positive cells and CD31-positive endothelial cells forming capillary-like ring-shaped structures (green cir-cles) within and near the HMSC fiber-derived tissue (Figure 8b-1′,b-2′ green circles and box). Vimentin staining further demonstrated a similar distribution of vimentin-positive mesenchymal cells in the surrounding thrombus (Figure 8b-2″), suggesting the involvement of mesenchymal cells, including myofibroblast-like cells, in fibroconnective tissue formation around the transplanted HMSC fiber. In contrast, fibroconnective tissue formation was scarce in aneurysms without HMSC fiber transplantation (Figure 8a-1′). α-SMA-positive cells were present within the HMSC fiber-derived tissue; however, most cells were α-SMA-negative and contained small, fragmented nuclei (white arrows), indicating cell death (Figure 8b-1′).

## 4. Discussion

The fundamental concept of EVAR is to prevent aneurysm rupture by stimulating thrombus formation within the aneurysm sac. However, thrombus formation alone does not facilitate the biological repair of the degenerated aneurysm wall. Previous cell therapy studies of aortic aneurysms have mostly been limited to small-animal models, in which the administration of MSCs via intravenous delivery or direct seeding has been reported to attenuate aneurysm enlargement [19,20,21]. While these findings support the therapeutic potential of MSCs, these studies evaluated cell therapy alone, overlooking the mechanisms of how MSC-based therapy might be functionally integrated via a type of endovascular treatment, which currently represents the clinical standard.

This study addressed translational gap arising from the absence of a catheter-deliverable cellular construct that can be stably retained within the aneurysm sac. To this end, we designed and engineered HMSC fiber as a scaffold-free cellular biomaterial that can retain endogenous ECM and enable precise geometric control. Using our CAT-based tissue fabrication technology [16], HMSC fibers were prepared to satisfy biological requirements related to cell viability, as well as procedural requirements associated with intravascular manipulation. CAT enables the spontaneous delamination and self-aggregation of cell monolayers without the use of exogenous scaffold materials, thereby facilitating the formation of three-dimensional constructs while preserving endogenous ECM. Consistent with this principle, HMSC fibers prepared in the present study retained ECM-rich, three-dimensional architectures, as shown in Figure 4C-1,C-2. Preservation of endogenous ECM represents a critical feature for regenerative applications, as it contributes to cell–cell adhesion, structural cohesion, and maintenance of the native cellular microenvironment [22]. In cell sheet engineering, temperature-responsive culture surfaces allow the recovery of cell sheets without enzymatic treatment [23] while preserving endogenous ECM. Furthermore, immunohistochemical analyses have confirmed the retention of ECM components in the harvested sheets [24]. Such ECM-retaining cell sheets can improve post-transplant cell survival and tissue engraftment [25] and have already been translated into clinical applications targeting tissues such as the cornea and myocardium [26,27].

From a procedural perspective, HMSC fibers maintained their morphology during forceps handling, as well as during aspiration into a catheter, without fragmentation or structural collapse (Figure 5, Supplementary Videos S1 and S2). This structural stability is attributable to strong cell–cell adhesion mediated by retained endogenous ECM, clearly distinguishing HMSC fibers from conventional cell suspension-based delivery approaches, which must be prone to dispersion and washout under arterial blood flow. Delivery of HMSC fiber as a single, continuous, shape-retaining construct enables localized and controllable cell placement within the aneurysm sac, thereby overcoming a major limitation of existing intravascular cell delivery strategies. Within the context of endovascular delivery, such scaffold-free and ECM-retaining architecture provides a structural basis for ensuring mechanical stability during catheter manipulation and for achieving high early-stage engraftment without introducing foreign biomaterials. Furthermore, by precisely controlling the number of cells per unit fiber length, we systematically investigated the relationship between fiber diameter and intra-fiber cell viability and identified a structural range that maintained sufficient cellular density while avoiding central necrosis (Figure 4B-1). This observation was further supported by quantitative viability analysis (Figure 4B-2), which demonstrated reduced cell survival at higher cell numbers per unit length, likely reflecting diffusion limitations within thicker fiber structures. Based on these findings, HMSC fibers prepared with 50–100 × 10^4^ cells/cm appear to provide a practical balance between cellular viability and structural stability. Fibers prepared at lower cell numbers tended to be thinner and mechanically less stable during catheter handling, whereas higher cell numbers resulted in reduced cell survival, likely due to diffusion limitations within thicker fiber structures. Further optimization of the cell number per unit length may improve both biological performance and handling properties for endovascular delivery. These findings indicate that HMSC fibers possess a defined design window in which biological constraints related to oxygen and nutrient diffusion and mechanical requirements for intravascular handling can be simultaneously satisfied. From a biomaterials perspective, this result highlights the importance of treating geometric structure itself as a key design parameter rather than merely considering cellular aggregates as size-variable assemblies.

The histological findings in the present study suggest an early tissue response that could be mediated, at least in part, by the paracrine activity of the transplanted HMSCs (Figure 8b-1′,b-2′), consistent with previous reports demonstrating that HMSCs promote angiogenesis via vascular endothelial growth factor and platelet-derived growth factor and contribute to tissue formation and ECM deposition through factors such as fibroblast growth factor and transforming growth factor [7,28,29]. Consistent with this mechanism, immunofluorescence analysis demonstrated VEGF-positive staining within the HMSC fibers prior to transplantation (Figure 4C-5), suggesting that VEGF secretion from the transplanted constructs may be associated with the observed capillary-like structure formation. In addition, vimentin-positive mesenchymal cells were observed within the thrombus surrounding the transplanted HMSC fiber (Figure 8b-2”), suggesting the involvement of HMSCs and/or host-derived mesenchymal cells in early fibroconnective tissue formation around the transplanted construct. A key limitation of this study is that transplantation was performed under a xenogeneic condition, where immune-mediated elimination of transplanted cells is unavoidable. Accordingly, sustained structural integration and long-term contribution to aneurysm wall reconstruction could not be fully evaluated. Future studies using allogeneic and longer-term models will be essential to clarify the relative contributions of paracrine signaling and direct tissue construction and to determine whether HMSC fiber delivery can contribute to durable aneurysm stabilization. Such studies will also be important to evaluate additional mechanisms underlying the therapeutic effects of HMSC fibers, including potential paracrine effects under immunologically compatible conditions, as well as the long-term consequences of neovascularization including the possibility of excessive fibroconnective tissue formation.

## 5. Conclusions

In this study, we prepared fiber-shaped HMSC aggregates that formed HMSC fibers optimized for an endovascular procedure by controlling the cell number per unit length. The resulting HMSC fibers retained MSC characteristics and endogenous ECM, facilitating stable handling and catheter-based transplantation. Following transplantation into a swine aortic aneurysm model, HMSC fibers induced fibroconnective tissue formation accompanied by capillary-like structures within the aneurysm sac. The results of this study demonstrate that HMSC fibers provide a controllable and stable platform for localized endovascular cell delivery, extending the concept of cell therapy beyond simple cell suspension injection. Further validation is required regarding the safety and efficacy of cell therapy for aortic diseases.

## Data Availability

The data supporting the findings of this study are available from the corresponding author upon reasonable request. Data are not publicly available due to ethical considerations related to animal experiments.

## Supplementary Materials

The following supporting information can be downloaded at https://www.mdpi.com/article/10.3390/jfb17040165/s1, Video S1: title; Handling of HMSC fiber using forceps. Video S2: title; Catheter loading of HMSC fiber.

## Abbreviations

α-SMA	Alfa-smooth muscle actin
CAT	Cell self-aggregation technique
CCA	Common carotid artery
ECM	Extracellular matrix
EVAR	Endovascular aortic aneurysm repair
HMSC	Human mesenchymal stromal cell
SG	Stent graft
TAA	Thoracic aortic aneurysm
